# The u-can-act Platform: A Tool to Study Intra-individual Processes of Early School Leaving and Its Prevention Using Multiple Informants

**DOI:** 10.3389/fpsyg.2019.01808

**Published:** 2019-09-20

**Authors:** Frank J. Blaauw, Mandy A. E. van der Gaag, Nick R. Snell, Ando C. Emerencia, E. Saskia Kunnen, Peter de Jonge

**Affiliations:** ^1^Department of Developmental Psychology, Faculty Behavioral and Social Sciences, University of Groningen, Groningen, Netherlands; ^2^Distributed Systems Group, Bernoulli Institute for Mathematics, Computer Science, and Artificial Intelligence, University of Groningen, Groningen, Netherlands

**Keywords:** early school leaving, ecological momentary assessments, web application, vocational education, motivation, open source

## Abstract

We present the u-can-act platform, a tool that we developed to study the individual processes of early school leaving and the preventative actions that mentors take to steer these processes in the right direction. Early school leaving is a significant problem, particularly in vocational education, and can have severe consequences for both the individual and society. However, the prevention of early school leaving is hampered by a mismatch between research and practice: research tends to focus on identifying risk factors using group averages and cross-sectional studies, while practitioners focus on intervening in individual processes. We aim to help solve this mismatch with our project *u-can-act*. In this project we have developed a platform that helps to gain insight into both the individual processes that precede early school leaving as well as the actions that mentors take to prevent it. In this paper we introduce the u-can-act platform, which consists of three technology-based, reusable methodological innovations. Specifically, our innovations concern: (i) an open source web application for longitudinal personalized data-collection, (ii) an automated study protocol that optimizes adherence in a difficult target group (adolescents at risk for early school leaving), and (iii) a technologically assisted coupling between mentor and student that allows us to study dyadic interactions over time. We present performance results of our platform, including participant adherence, the behavior of the questionnaire items over time, and the way that our web application is experienced by the participants. We conclude that our innovative platform is successful in collecting multi-informant time-series data on intervention processes among students in vocational education, both for at-risk students and control students, and for their mentors. Moreover, our platform is suitable for broader applications: it can be used to study any malleable individual process including the efforts of a second individual who aims to influence this process. Because of the unique insights that the u-can-act platform is able to generate, the platform may ultimately contribute to solving the mismatch between research and practice, and to more effective interventions in individual processes.

## 1. Introduction

Each year, many adolescents and young adults leave school early^1^. In Europe alone, 5.5 million individuals left school early in 2012 (European Commission, 2013). Early school leaving is a particularly large problem in vocational education and training (VET), where approximately two thirds of all European early school leaving takes place (Cedefop, 2016). This is alarming as early school leaving has severe consequences for both the individual and society as a whole. For example, compared to individuals who obtain a starting qualification, early school leavers have a weaker position on the labor market (e.g., a higher risk of unemployment, lower income, more precarious work conditions) and experience less health, a lower life expectancy, and less life satisfaction (Cedefop, 2016).

Thus it comes at no surprise that on the one hand scientists have spent much effort to investigate the causes and consequences of early school leaving and on the other hand practitioners have spent much effort to try to prevent early school leaving. However, the efforts of the practitioners are not always optimally informed by science. This sub-optimal information may be due to a mismatch in focus: while social scientists have a long tradition of generating knowledge on the *between-individual* level (e.g., finding general trends based on data retrieved from groups), practitioners tend to focus on the *within-individual level* (i.e., individual change processes). This mismatch has two important consequences.

Firstly, our body of between-individual scientific knowledge has facilitated the identification of individuals at risk for early school leaving but has hardly informed prevention strategies. For instance, it has been shown that early school leaving is more likely to occur among males, individuals with a migration background, and individuals with a low social economic status (Rosenthal, 1998). Although this general information is valuable for identifying at-risk individuals, it has little utility to steer interventions of a practitioner, as it is impossible for a practitioner to adjust these factors. Other, more malleable factors have also been demonstrated to be risk factors for dropout, such as problem behavior or negative attitudes toward school (Rumberger and Lim, 2008). Even though a focus on malleable factors is already more useful to the practitioner, merely focusing on malleable factors is still too limited, as reducing risk factors is not the same as promoting graduation and positive youth development (Zaff et al., 2017). In order to perform such promotion, more knowledge is needed on how within-individual processes of positive, malleable factors that are known to promote graduation, such as motivation and engagement (Zaff et al., 2017), can be directly affected by practitioners who work with adolescents.

Secondly, it is fundamentally ill-advisable to use between-individual knowledge to inform within-individual processes. Although research on the between-individual level can provide general information about group characteristics, it provides knowledge that is true on average, but that might not hold true for any individual in specific (e.g., *the non-existent average individual*; Allport, 1937; Blaauw, 2018). Moreover, between-individual knowledge may obfuscate the relations on the individual level, meaning that findings on the between-individual level may not exist on the within-individual level, and can indeed even be opposite (e.g., *Simpson's paradox*; Simpson, 1951; Blyth, 1972, *the ecological fallacy*; Piantadosi et al., 1988, and *non-ergodicity*; Molenaar, 2004; Hamaker, 2012). These problems with translating between-individual findings to within-individual processes are thought to be relevant for the majority of psychological processes (Molenaar, 2004; Kievit et al., 2013). As such, in order to inform practitioners on the individual processes of early school leaving, and how to steer these in the right direction, within-individual research is a necessity.

Fortunately, technological developments have made it increasingly feasible to study within-individual processes. A prominent method to do this is the Ecological Momentary Assessment (EMA) methodology, also known as the experience sampling methodology (ESM), or diary studies (Csikszentmihalyi and Larson, 1987; Shiffman and Stone, 1998). EMA is a methodology widely used in psychopathology research and behavioral research (e.g., Bolger et al., 2003; Trull and Ebner-Priemer, 2009; van der Krieke et al., 2016a). In an EMA study, a participant completes the same questionnaire for a long period of time, possibly multiple times per day, resulting in a large number of measurements of multiple (psychological) variables within one individual. This type of high resolution dataset can provide insight into the processes of the measured variables over time, and the relations between them, within a specific individual. Moreover, the data about an individual can be used to shed light on intra-individual variability, which would be unknown (or assumed non-existent) in a cross-sectional study.

In this paper we present the open source EMA platform of the *u-can-act* research project that we use to study the developmental processes of early school leaving in students, their micro-level interactions with their mentors, and the prevention of early school leaving within individuals. The platform aims to help researchers to effectively study dynamic within-individual processes from multiple informants, even among difficult to reach target groups. It does so by providing an automated way for collecting longitudinal questionnaire data and managing the connections between different informants. The platform can be reused and adapted by other researchers because it is fully open source. The platform is shaped by the aims and theoretical foundations of the u-can-act project, which we present in section 2. The platform encompasses three technological innovations that we present in-depth in section 3, these concern (i) the development of an open source EMA application, (ii) the development of an automated EMA protocol that aims to maximize adherence, and (iii) an innovative coupled multi-informant setup that enables us to investigate dyadic interactions as dynamic processes over time. We collected data among students and their mentors, described in section 4 and use this data to present findings on the performance of the platform. In particular, we focus on its ability to capture within-individual dynamics, the ease of participation for both mentors and students (including early school leavers) and the usability of the platform in section 5. We conclude that our platform is successful in achieving its aim and provide direction for future studies in section 6.

## 2. Theoretical Foundations and Aims of u-can-act

The u-can-act platform and its technological innovations (see also section 3) have their current form because of the aims and the underlying theory that we use in the u-can-act project to study early school leaving and its prevention. U-can-act focuses on (i) malleable, dynamic factors that are relevant to early school leaving (section 2.1) and (ii) dynamic within-individuals processes and dyadic interactions on a weekly, micro-level time-scale (section 2.2). This allows us to clarify processes that precede early school leaving and determine the effects of the mentors' preventative actions on the development of the student, and ultimately, on the students' early school leaving intentions. With this information we aim to inform practitioners on a very practical and detailed level on the actions to take and when to take them, and help policy makers to choose preventative strategies that seem beneficial in reducing early school leaving. We have translated these aims in a theoretical model that reflects our main assumptions (section 2.3). This theoretical model forms the basis of our u-can-act platform.

### 2.1. A Focus On Malleable Factors

We focus specifically on malleable factors that are expected to vary over time within individuals, and that have the potential to not only prevent early school leaving, but to also promote positive development. A central theory we use for this is the *self-determination* theory. Self-determination theory is an important aspect of the process of early school leaving, while at the same time it is also an important means to promote positive development and intervene in the process of early school leaving (Vallerand et al., 1997; Zaff et al., 2017). The self-determination theory, as proposed and investigated by Deci and Ryan (2012), is primarily a theory of motivation. It postulates the existence of three basic psychological needs, which are autonomy, relatedness, and competence. The fulfillment of basic psychological needs fosters intrinsic motivation, but has recently also been ascribed a broader function: Deci and Ryan (2012) describe that the fulfillment of these needs is “essential for optimal development and functioning” (p. 417). Indeed, need fulfillment has empirically been related to many indicators of well-being and growth, while the frustration of needs is related to illbeing and maladaptive functioning (Vansteenkiste and Ryan, 2013), and of course, early school leaving (e.g., Hardre and Reeve, 2003; Alivernini and Lucidi, 2011).

The interesting characteristic about psychological needs is that they are changeable and can be supported (Hardre and Reeve, 2003; Ntoumanis, 2005; Mouratidis et al., 2011)—thus they form a particularly interesting source of information for practitioners. In fact, in a Dutch study that investigated fifteen early school leaving prevention and intervention projects it was found that the large variety of approaches could be uniformly characterized as aiming to support the autonomy, competence, and relatedness of the students (Heemskerk et al., 2018).

Besides need fulfillment, we focus on two other malleable variables relevant to early school leaving: engagement and expected success. Engagement is an important, malleable factor in the process of early school leaving (Fredricks et al., 2004) and can be defined in several ways (Nielsen, 2016), we chose to focus on two of these. First, behavioral engagement, which is a form of engagement that emphasizes involvement in activities, and is considered crucial in attaining positive academic outcomes and preventing dropout (Fredricks et al., 2004). This is perhaps the most commonly studied form of engagement, but has also been criticized to be one-sided and behavioristic (Nielsen, 2016). Therefore we also study emotional engagement, which has also been found to be an important predictor of early school leaving, besides behavioral engagement (Wang and Fredricks, 2014). In addition to engagement, we focus on the expectations that the students have about the academic success that they will obtain during the school year, as such expectations have also turned out to be malleable yet important predictors of persistence and school success (Zaff et al., 2017).

### 2.2. A Focus On Individual Processes On a Micro-Level

Much is still unknown about psychological need fulfillment and engagement as part of within-individual, micro-level processes that may change over a short time-span, like weeks or even days. However, some first steps have been made, for example by van der Kaap-Deeder et al. (2017). They found that a sense of autonomy satisfaction or frustration was directly influenced by daily interactions. Moreover they found that the social contexts of these interactions matters, as each of the three social contexts they studied (interactions with mothers, teachers, and siblings), uniquely contributed to whether autonomy satisfaction or frustration is experienced.

Thus experiences in different contexts have the potential to either fulfill or frustrate psychological needs and a within-individual approach is necessary to understand the long-term consequence that this may have for early school leaving. For example, Aelterman et al. (2016) propose that need fulfilling activities result in a pull on the individual, attracting the individual to spend energy on the target activity, while need thwarting activities push the individual away. Extending this hypothesis, we can imagine that in some individual cases need fulfillment may in fact increase the chance of dropout: when individuals spend all their time outside of school because of the need fulfilling context, their engagement with school may decrease and dropout may eventually follow. Such a hypothetical process contradicts the common group-finding that need fulfillment is generally beneficial (Vansteenkiste and Ryan, 2013) and remains unexplored in studies so far because of their inter-individual focus (see also section 1). We can only gain insight into the existence of such hypothetical individual processes by taking a within-individual approach.

Moreover, a micro-level, within-individual approach is necessary in order to learn more about the role that mentors play in influencing students' development and preventing dropout. Individual guidance has proven to be quite effective to prevent early school leaving in many independent intervention and prevention programs (Heemskerk et al., 2018), but much is still unknown about the ingredients of such guidance. Which concrete actions do mentors take in their guidance of adolescents, what goals do they strive for? How effective are they in supporting the basic psychological needs of their students from week to week? Such questions can only be answered by studying the within-individual guidance processes of mentors and the micro-level interactions between students and mentors.

### 2.3. The Theoretical Foundations of the u-can-act Platform

**Figure 1:**
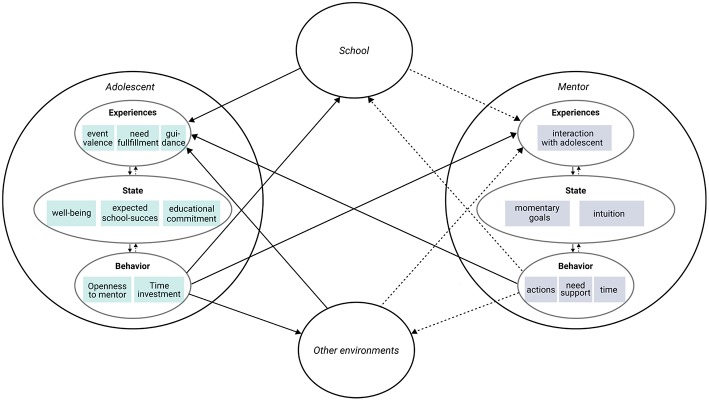
Hypothesized theoretical model of early school leaving that we use in the u-can-act project. In future studies, our platform allows us to study the hypothetical empirical relations that are indicated with a solid line (the relations indicated by the dotted lines cannot be studied in our current set-up).

We placed the malleable factors relevant for early school leaving in a hypothetical model that reflects our within-individual process approach (see Figure 1) and have used this model to as the foundation for the u-can-act platform. The interplay between the student and different contexts is at the heart of our theoretical model. Indeed, our within individual approach has led us to hypothesize that the interplay of need fulfillment inside and outside of the school is an important process underlying early school leaving, while it is at the same time a process that a mentor can potentially influence in order to prevent early school leaving. Because we are particularly interested in informing mentors on what they can do to help prevent dropout, the student-mentor interaction is central in our model.

Figure 1 schematically represents the hypothetical model. It reflects the main theoretical assumptions that have driven the development and innovations of our dual informant EMA-platform, and includes the measures that we have employed. These measures cover different aspects of individuals' experience, mental state, and behavior, that are hypothesized to be relevant for the process of early school leaving and interventions in this process. Perhaps the most important assumption that is reflected by this theoretical model, is that students continuously interact with several environments, including a school environment, other environments (non-school, such as the home environment) and their mentor. We included the students' experiences of events and need fulfillment in both school and non-school contexts, as well as experiences of mentor need support and quality of the guidance they receive. We operationalize the students' mental state as emotional engagement, current school success expectations, and well-being. We measure the students' behavior by assessing the amount of time they spent on school activities and how open they have been with their mentor. Similar to the students, mentors have experiences, mental states, and behavior as well, which we operationalized solely with variables relevant to the student-mentor interaction. We assume that the mentors can experience various degrees of satisfaction in their interaction with the student. As a mental state, they can also have various degrees of intuitiveness when performing their actions (as opposed to performing planned actions), and have certain goals they want to achieve. Ideally, their goals are reflected in their actions, but also in their support of students' needs and in their time-investments in the student. This mentor-behavior can be perceived by the student in the quality of the guidance and in the support he or she feels in need fulfillment, with which the student-mentor interaction cycle has come full circle.

To test the relations and processes in our hypothetical model we needed a suitable measurement instrument that met at least three requirements. First and foremost, the instrument needed to repeatedly measure individuals over a period of time in order to gain insight into the within-individual dynamic processes of early school leaving. Secondly, the instrument needed to optimally facilitate easy participation, in order to gather enough data. After all, the processes of motivation that could underlie early school leaving might also influence the motivation of students to partake in this study. Thirdly, the instrument needed to be able to collect measurements for both students and their mentors in order to gain insight into their interaction and into the actions that mentors can take in order to prevent early school leaving. For this, a coupling between the two measurements was necessary. Because there were no applications available that met these requirements, we set out to develop such an application: the u-can-act platform.

## 3. The u-can-act Platform

We developed a platform that allows for studying within-individual processes and dyadic interactions within an intervention setting, from a multi-informant perspective. The platform is rooted in three technological innovations.

The first innovation, and the foundation of our data-collection, is the development of a web application that applies a fully automated method for scheduling, sending invitations, and hosting EMA questionnaires. This free and open source application provides participants with a web interface to fill out weekly questionnaires. Our second innovation is a study protocol that optimizes participant adherence among a difficult target group, which includes an elaborate reward system and messaging that is automatically adapted to the participation behavior of each individual participant. The third innovation is the development of a multi-informant EMA questionnaire that allows us to study the process of early school leaving and the preventative actions in this process from both the student and mentor perspective, where the technology behind our platform manages and deals with the multi-informant aspect of our study by automatically coupling the mentors to their students. We will introduce the three innovations in more detail below.

The three innovations are all integrated in one open source software package, developed by Emerencia et al. (2017) and is freely available at http://u-can-act.com.

### 3.1. Innovation 1: An Open Source Web-Application

Our first innovation is perhaps most fundamental to our approach: an open source web application that measures the developmental processes of students and their micro-level interactions with their mentors. The application schedules and sends out questionnaire invites automatically, and stores the data inside two separate and secure databases (one containing personal data and one containing the answers to the questionnaires). Screenshots of the application can be found in the Supplementary Material in Figure S1.

**Figure 2:**
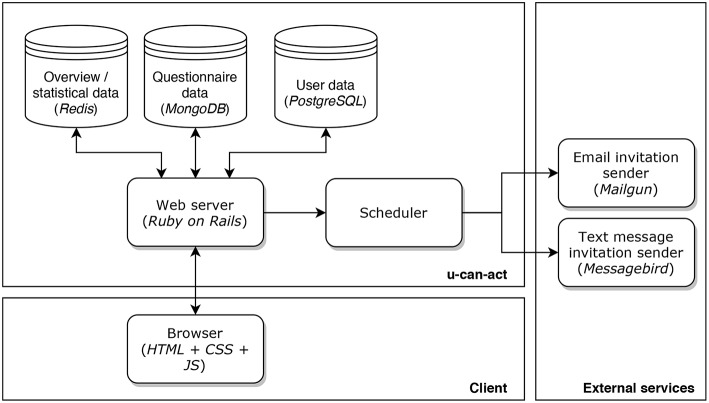
Technological infrastructure of the u-can-act web application.

A schematic overview of the technological infrastructure of the u-can-act platform is provided in Figure 2. The platform serves its content by means of a web application implemented in the Ruby on Rails framework. Ruby on Rails is an open source framework that provides a default structure for web applications^2^. In order for other researchers, schools, and agencies to be free to use and adapt its implementation, we released u-can-act as MIT-licensed^3^ open source software on https://u-can-act.com. The implementation of u-can-act builds upon our experience in designing architectures for web-based questionnaire platforms, such as the implementation of the HowNutsAreTheDutch web application (Blaauw and Emerencia, 2015; van der Krieke et al., 2016b).

The collected data is stored into two separate databases: one database that holds the questionnaire data, and one database that contains all personal data. The latter database keeps track of the completed questionnaires by storing a reference to the actual questionnaire data. This separation ensures anonymity in case of a breach in one of these databases. The personal information is stored in a relational SQL database named PostgreSQL. The questionnaire data is stored in a MongoDB NoSQL database. The rationale behind the choice for MongoDB is that it provides a schemaless document storage, which fits well with storing different types of questionnaire data. Finally, we use a third Redis NoSQL database that contains the aggregated / analytical data for caching purposes. Data stored in this database are considered volatile, and mostly used on the researcher dashboard, to provide them with general statistics about the questionnaire completion percentages and rewards collected. Without this cache, these data need to be calculated in real time, which negatively influences the performance of the application.

The traffic to the web application is protected using a 2048 bit RSA (SHA 256 bit) TLS 1.2 Secure Socket Layer (SSL) connection, which ensures private data exchange to and from the u-can-act web application. The interactions with the underlying database infrastructure are protected using SSL as well. Filling out a questionnaire is only possible via the link sent to the participant in a text sent to their phones or in an email message. These links contain a user identifier and a token. The tokens are stored using the Bcrypt encryption standard, which makes it practically impossible to retrieve the clear-text token from its encrypted counterpart.

The platform is built as generic, reusable software, such that other research projects could reuse the platform. Areas in which this could be of interest are, for example, psychiatry (e.g., HowNutsAreTheDutch and Leefplezier; Blaauw et al., 2014a,b; van der Krieke et al., 2016a), general health (Nair et al., 2016), pain monitoring (Stone et al., 2003), substance abuse (Shiffman, 2009), and many other fields that benefit from intra-individual measurements.

The u-can-act application automatically schedules questionnaires and invitations for each participant in the system. During the initial setup phase, the u-can-act application is initialized with a definition of the protocol that contains the collection of measurements, the interval at which invitations should be sent, and the actual questionnaire items that need to be completed. Subsequently all participants can be subscribed to their protocols at any given start and end date. The u-can-act application automatically invites them to complete their questionnaire on a given interval by means of a text message or email.

### 3.2. Innovation 2: Optimizing Adherence and Study Load

Most students included in this study have a high risk of early school leaving, which might also be a risk for their participation behavior in the u-can-act study. Hence, optimizing the study adherence and minimizing the study load has been an important priority for u-can-act. As such, we performed three adherence-optimization steps, which were largely informed by our initial pilot study.

Firstly, we determined an EMA schedule that would work optimally for our sample. From our pilot study, we concluded that the optimal measurement interval is once a week for both the students and the mentors. The main reason for selecting this measurement interval is threefold: (i) this interval coincides well with the frequency of the meetings between student and mentor, (ii) this measurement interval did not significantly reduce the variance in the items compared to more frequent intervals that we also included in the pilot study, and (iii) the evaluation results showed that participants expected this study interval to be most sustainable.

Secondly, in our pilot study we performed interviews, observational studies, and a detailed analysis of each questionnaire question to optimize the users' experience and minimize time-investment while using the application. We optimized the questionnaire questions that scored lowest on understandability and incorporated many qualitative recommendations to increase the usability of the app. This involved, for example, reformulating questions to ask about concrete categories (instead of free text, broader categories, or actions), and providing more information about the meaning, context, and purpose of questions.

Thirdly, and perhaps most importantly, the qualitative data from the pilot study and brainstorm sessions within both our team and one of the involved guidance agencies informed our intrinsic and extrinsic motivational strategies, which we will describe in more detail below.

#### 3.2.1. Fostering Intrinsic Motivation: Personalization

The students receive one SMS text message per week for approximately 35 weeks during the study to inform them that the questionnaire is available for them to fill out. The text messages are framed in a positive way, emphasizing the value of their contribution for their mentor and the research project. The contents of the text messages were dynamically constructed and personalized for each user, taking into account the participation figures (see Figure S3 in the Supplementary Material for an overview of the invite message and personalization procedure). The rationale behind sending different and personalized text messages was that both the fact that the message text was variable and that it was personalized potentially has a motivating effect for actually filling out the questionnaire (Heerwegh et al., 2005; Muñoz-Leiva et al., 2010).

A second personalization step was performed in the questionnaires themselves. U-can-act uses a system that can automatically tailor questionnaires toward the individual. This means that certain variables are replaced with values relevant to the participant. For example ‘your mentor' would be changed to the actual name of the mentor. The options that were available for personalization were: (i) the name of the mentor, (ii) the name of the student, (iii) the gender of the student (different forms), and (iv) the name of the supervisory agency they were affiliated with.

#### 3.2.2. Fostering Extrinsic Motivation: Monetary Rewards for Students

After the EMA study was completed, students received a monetary reward that reflected their amount of completed questionnaires. They received a two Euro reward for each questionnaire they completed. If students completed three questionnaires consecutively, they were awarded a so-called “bonus Euro,” which was an additional one Euro reward on top of the two Euro reward. This bonus Euro is an example of *gamification* and aims to motivate the students to complete longer questionnaire series and not leave many gaps, which can be troublesome for certain analyses. The bonus Euro was awarded for each completed questionnaire until one questionnaire was missed, after which the students again needed to complete three consecutive questionnaires. After each completed questionnaire a reward page was displayed to the students. On this page they could see the monetary rewards that they had already earned, the rewards that were still earnable, their progress toward the end-goal (the maximum amount of reward) and their bonus streak. All this was displayed using a playful design, see Figure S1 in the Supplementary Material for a visualization.

### 3.3. Innovation 3: Multi-Informant EMA to Study Students, Mentors, and Their Interactions

The u-can-act platform maps out the process of early school leaving and preventative actions in this process from two perspectives: students and mentors. On the one hand, u-can-act collects weekly data about students and their own experiences. On the other hand, the platform takes the perspective of the mentors into account, by asking them to complete questionnaires for each of the students that they supervise. The database is set up in such a way that an automatic coupling is made between each student and their mentor, which enables us to study the interactions between them. Moreover, this coupling helps foster personalization (see also section 3.2), as for example, students see their mentor's name when answering questions about the quality of his or her supervision. We provide more detail on the data collection among students and mentors in sections 4.2 and 4.3.

## 4. Methods for Evaluating the Platform

We collected empirical data among students and mentors during the u-can-act project that we use to evaluate the performance of the u-can-act platform and its three innovations. For this evaluation, we check whether the platform meets three requirements (see also section 2.3) that we believe are essential in order to measure within-individual dynamic processes among adolescents and the interactions with their mentors: (i) dynamicity of the measures, (ii) easy participation, and (iii) good user experience. We describe our data-collection protocol and measurement instruments for both the student as well as the mentor study and give a brief description of our methods for analyzing the performance of the platform.

### 4.1. Ethics

The u-can-act research protocol was assessed and approved by the ethical committee of the University of Groningen under code 16351-O. All participants provided their informed consent online. No explicit informed consent was collected from the parents/legal guardians of non-adult participants, as all participants were above the age of sixteen.

### 4.2. Student Study

**Figure 3:**
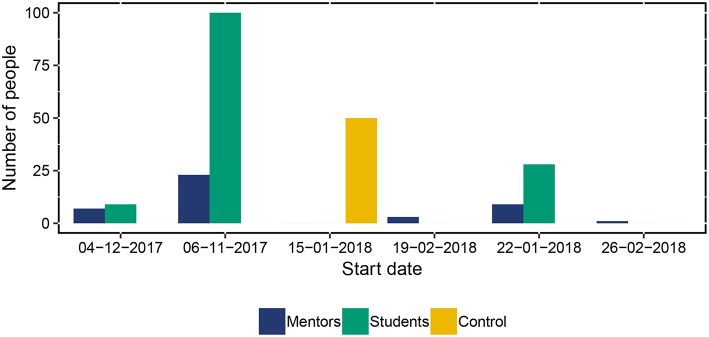
Dates of enrollment of students and mentors.

The first students were enrolled in the student study on November 6, 2017. Students and mentors joined the study on six moments, for an overview see Figure 3. The students that participated were all participating in secondary vocational education in three locations spread throughout the Netherlands. The students that participated in this study could be in one of two sub-groups: an *at-risk* subgroup, or a *control* subgroup. The students in the at-risk subgroup were considered to be at risk of early school leaving by their own educational institution, for example because their grades were low, they attended only few classes, experienced stressful situations at home, or showed disruptive behavior in class. Because of this elevated risk, these students were signed up for extra individual guidance. The individual guidance was supplied by mentors from three different supervision agencies (more on this in section 4.3). We approached the students through their mentors: we first asked the mentors to participate, who then asked their students to participate. The students in the control subgroup did not have a mentor, as they were not considered to be at risk for early school leaving and were approached directly.

The student study comprises three main questionnaires: (i) a general assessment, (ii) an EMA questionnaire, and (iii) a post-assessment. The general assessment collected information about the students' demographics and living situation. The EMA questionnaire collected information on variables that could fluctuate over time and are hypothesized to potentially underlie early school leaving (i.e., autonomy, competence, and relatedness). The post-assessment collected information about their current educational situation, such as whether they were still enrolled in their educational track, and whether they intend to complete the track.

#### 4.2.1. Procedure

In order to participate in the study, a student had to be subscribed to the u-can-act platform and provide online informed consent. The control subgroup students were randomly selected from one educational institution in the Northern part of the Netherlands. In collaboration with this educational institution we sampled several students that were considered to be not be at risk of early school leaving, and had not received additional supervision from within their educational institution to help them with school or private problems. If they agreed to participate and accepted the informed consent, they were enrolled in the study.

All students in this study followed the same assessment protocol. Near the start of the EMA study, students were asked to complete a required general assessment questionnaire. Then, for approximately 35 weeks (or until the beginning of the summer holiday period, whichever was shorter), they received a personalized text message each Thursday at noon, in which they were requested to fill out a questionnaire. Each text message contained a link to the u-can-act web application that provided access to the questionnaire they had to fill out. The application automatically sent a reminder text message 8 h later in case a student did not complete the questionnaire before that time. Questionnaires were available for 30 h after the initial invitation. To facilitate early stopping from the study, students were presented with a button with which they could unsubscribe from the study on June 28, 2018. This button presented them with the question whether their summer holiday had already started, and if it did, that they could end their subscription now, after which they would receive a final, post-assessment questionnaire.

#### 4.2.2. Student Questionnaire Items

The general assessment consisted of nine questions, with which we collect data about (i) birth year, (ii) nationality, (iii) relationship status, (iv) whether or not they had children (and how many), (v) the name of the school they attend, (vi) the type of education they follow, (vii) the level of education, (viii) how many years of education they followed thus far, and (ix) what the students did before starting their current studies. The full list of questions and corresponding answer options is presented in the Supplementary Material in Table S1. We collected data about gender during the sign-up process, along with first name, last name, and mobile phone number.

The weekly student EMA questionnaire consisted of twenty-five questions. These questions were in most cases newly created for the purpose of this study, or adapted from previous questionnaires. All questionnaire items are described in Table S2 in the Supplementary Material. The questionnaire items were selected to assess experienced autonomy, competence and relatedness in three contexts (school, outside-of-school, and mentor relationship); behavioral and emotional engagement with school; school success expectations; evaluations of their mentors' actions; their general level of well-being and the general valence of their school experiences. An interactive example of the web application can be found online^4^. Note that for the control group, all questions related to supervision of a mentor were removed as they were not applicable (questions 18–24).

The visual design of the questionnaire is composed of three different question options: (i) visual analog scales (VAS), (ii) radio buttons, and (iii) checkboxes. Each of the VAS scales provides a continuous value ranging from 0 to 100, and displays a small indicator showing the selected number. The default value of the VAS scale was set to 50 (the center of the scale), and the extremes of the scale had appropriate labels (e.g., “not at all” to “very much,” see Table S2, “Response range”). The checkboxes and radio buttons were used to create multiple choice questions of which, respectively, multiple or only a single answer could be selected. In some cases, the radio questions had an option which allowed for the input of free text.

The post-assessment questionnaire consisted of at least 11 and at most 14 items (depending on the answers to the questions). The questionnaire focused on (i) whether the student dropped out or not (and when), (ii) the average grade of the student, (iii) if the students dropped out we asked whether they would start a new study and if the students persisted, how certain they are that they will complete this study, (iv) their average grade, (v) the quality of the supervision of the mentor, and (vi) some general questions related to the evaluation of the web application. The full questionnaire is provided in Table S3 in the Supplementary Material.

### 4.3. Mentor Study

The mentor study started at the same date as the student study, November 6, 2017 (see Figure 3 for more information), and consisted of three personal self-report questionnaires: a general assessment, a post-assessment and a series of EMA questionnaires about the students that they supervise. Each mentor completed diary questionnaires about their mentoring of each of their students separately. As such, the mentors essentially participated in several parallel EMA studies, one for each of their students.

#### 4.3.1. Procedure

The enrollment procedure for mentors was similar to the student enrollment procedure, although mentors could only participate whenever the mentor was actually actively involved in the supervision of one or more students. We asked the mentors to provide some general, personal information in a general assessment questionnaire. This general assessment questionnaire consisted of four questions concerning (i) education level, (ii) year of birth, (iii) years experience in supervising students, and (iv) nationality. The questionnaire and its items are listed in Table S4 in the Supplementary Material. Note that the gender for each participant was already known upon sign-up.

Similar to the student study, mentors received a weekly text message on Thursday around noon. In addition to the text message, mentors also received an email. Both the text message and email contained an invitation text and a link to their mentor dashboards, which provided each mentor with an overview of the questionnaires they had completed for the students that week and the adherence of each of their students by means of a heat map. This information could be used by the mentors to intervene if too many measurements were missed by a student. An illustration of this dashboard is provided in Figure S2 in the Supplementary Material. The mentors did explicitly not have access to the actual questionnaire data provided by the students, in order to provide anonymity to the students.

An interactive version of the mentor dashboard and mentor questionnaire is available online^5^. The system automatically reminded the mentors via e-mail and text message to fill out all the questionnaires if they had not done so 8 h after the initial invite.

At the end of the study, or after the mentors clicked a button telling the system that their holiday had started, a mentor received a post-assessment questionnaire. The post-assessment had a dynamic number of items, depending on the number of students that they supervised. It contained six questions related to their experience with supervising students, general questions related to the web application, and one question for each of the students they supervised, asking whether and by how much the student has improved during the supervision phase. The full list of questionnaire items for the post-assessment questionnaire is provided in Table S7 in the Supplementary Material.

#### 4.3.2. Mentor Questionnaire Items

The mentor EMA questionnaire was constructed in a bottom-up fashion: we designed the instrument in several brainstorm and focus-group sessions with one of the mentoring initiatives. An important outcome of these sessions was a categorization of the actions and goals that mentors frequently take in their guidance of students. In this way we aimed to measure variables that are highly relevant to the mentoring process. All mentor questionnaire items are listed in Table S6 in the Supplementary Material.

The mentor questionnaire was different from the student questionnaire in the sense that it was partly dynamic based on the needs of the mentor and could consist of a varying number of questions. By default, the questionnaire contained 24 questions, which could dynamically be extended to a maximum of sixty-nine questions depending on the information a mentor wanted to provide. The dynamic part of this questionnaire resides in its third question, which reads “Add another action (or series of actions).” This question provided the mentors to add up-to ten new action clusters (see Table S6 in the Supplementary Material) to record actions they had performed for the current student.

### 4.4. Analysis of the Platform

We show whether our platform indeed captures within-individual dynamics by calculating the root mean squared successive difference (RMSSD) for each of the questions in the separate questionnaires. The RMSSD is a measure of instability, and provides insight into the fluctuation of a variable over time (von Neumann et al., 1941). Fluctuation or variability is important for questions to be meaningful in an EMA (if a question does not fluctuate, there is no value in repeatedly collecting it) and is necessary to capture in order to gain more insight into within-individual processes over time. We calculate the average RMSSD of each of the continuous variables for each participant in separation and then report the average.

Next, we provide insight into the ease of participation by firstly providing a detailed overview of the adherence to the study over time for all the followed subgroups. We zoom into the adherence among students who dropped out of their educational trajectory. Secondly, we show how long it takes to fill in the questionnaire. We have implemented a questionnaire system that records the difference in time between subsequent questions in the questionnaire that allows us to do so. These timings provide a general insight into the ease of answering questions, and into which questions take more time than others and might be candidates for revision in future research.

Finally, we give some preliminary insight into whether our platform is able to take successful measurements among both students and mentors. We do so by reporting on how our participants have experienced the use of our platform using quality indicators from the post-assessment. Specifically, we asked all participants to grade the application on a scale from 1 to 10 (in steps of 0.5). Furthermore, we asked how difficult they found it to keep participating in the study on a scale from 0 to 100, where 0 denotes that it was very difficult to participate and 100 denotes that it was easy to participate.

## 5. Results

Before we evaluate the platform we first provide some characteristics of our sample. We then evaluate the performance by demonstrating the dynamics of the items, the ease of participation and the user experience of the platform.

### 5.1. Sample Characteristics

On July 27, 2018 the data collection in the u-can-act project was completed. The data set comprises of a total of 40 mentors from three supervisory agencies that participated in u-can-act, and 181 students, of which 50 are in the control group. We excluded one participant from the dataset because of seemingly unrealistic answer patterns; this individual had left all the sliders on their default value, without manually placing them there. Moreover, we found that individuals with older browsers did not see some questions (hidden questions that were toggled by other questions). This error occurred in 4.9% of the data, which was also excluded. The application was fixed to resolve this error in future studies.

The mean age of the mentors was 33.09 years (median = 28, range 20–49, standard deviation [SD] = 12.62) and 67.44% were women. The mentors had on average 4.46 years of experience (median = 2, range 0–25, SD = 5.96). Most mentors (95.35%) had the Dutch nationality. 83.33% of the mentors had at least finished intermediate vocational education.

In the at-risk student sample, the mean age was 20.59 years (median = 20, range 16-33, SD = 2.63) and 54.74% were women. The control student sample had similar characteristics and were on average 19.17 years old (median = 19, range 17–25, SD = 1.92) and contained slightly more women (66%). The students started their current study after: high school (at-risk: 39.2%, control: 70.83%), another secondary vocational education trajectory (at-risk: 45.6%, control: 20.83%), working (at-risk: 4.8%, control: 6.25%), or something else (at-risk: 10.4%, control: 2.08%).

**Figure 4:**
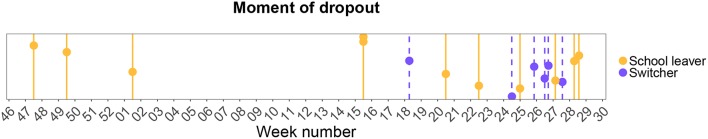
The week of the year in which people dropped out or switched to another study. Each dot is an individual who dropped out.

There were 17 students who dropped out of their educational trajectory during our study. All of the dropouts were in the at-risk group and none were in the control group. Of these dropouts, 11 left the educational system entirely (“school leavers”), while 6 students had plans to switch to a new educational trajectory (“switchers”). Most of the students, particularly the switchers, dropped out near the end of the academic year in the Netherlands (which coincided with the end of our measurements). For an overview of the dropout moments see Figure 4.

### 5.2. Dynamics of the Questionnaires Items

We calculated RMSSD's to investigate whether our instrument was capable of capturing the dynamics of within individual processes. Over all groups and items, the average RMSSD was 16.22 (median = 13.72, range 7.85–68.47). We also calculated RMSSD's for each of the item separately, these are listed in the tables describing the questionnaire items (Table S5 for the mentor questionnaire and Table S2 for the student at-risk and control questionnaire). The RMSSDs indicate that most items showed, on average, reasonable variation, and that none of the questions had drastically more variation than the others. The outlier of question 4 in Table S5 can be attributed to the fact that this question asked for the time spent on the supervision of a student, and is therefore scaled differently than the other questions, which are ranged 0–100.

### 5.3. Ease of Participation

Ease of participation was measured by both global adherence numbers (i.e., the number of filled out questionnaires) and the time it took for each questionnaire to be completed.

#### 5.3.1. Adherence to the Study Protocol

Across all agencies, the participants completed a total 6659 assessments On average, each at-risk student that started the diary study^6^ completed approximately 68.25% of their possible diary questionnaires. The control group completed approximately 83% of their possible diary questionnaires. The completion rate of the mentors was slightly lower at 52.28%.

**Figure 5:**
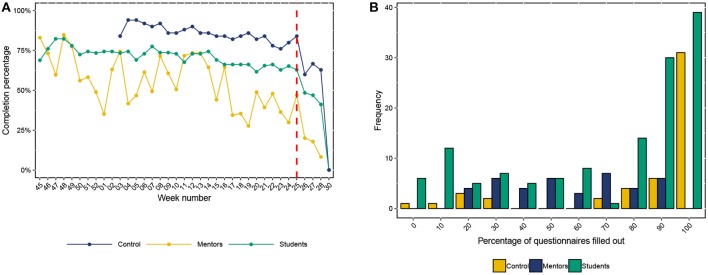
Study adherence for each subgroup. **(A)** Questionnaire completion over time in percentages. The red line indicates when participants could unsubscribe. **(B)** Cross-sectional questionnaire completion in percentages.

The adherence to the study over time is depicted in Figure 5. Here, Figure 5A shows the general (normalized) adherence to the study over time, in terms of completed questionnaires per group (control students, mentors, and at-risk students). Participation dropped rapidly after week 25, probably because we provided the participants with the option to finish their participation and fill-out the post-assessment questionnaire, as summer vacations started for many of them. In Figure 5B, we show the distribution of the percentage completed questionnaires for each of the subgroups. It is interesting to note that most of the at-risk students and the students in the control group completed at least 90% or even 100% of the questionnaires, while only a small part of the mentors showed such consistent adherence.

**Figure 6:**
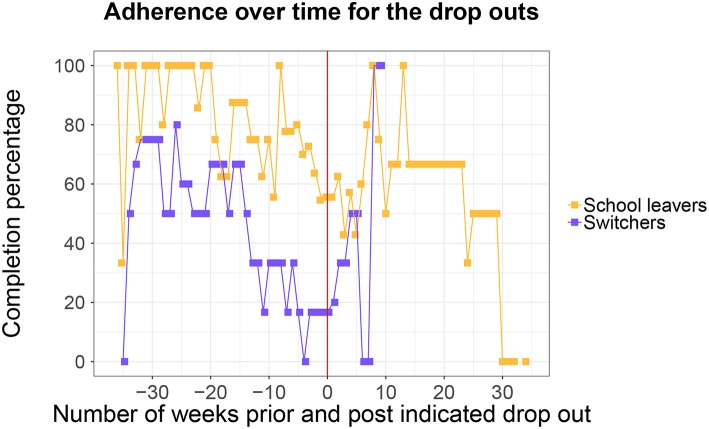
The number of completed questionnaires for the switchers and school leavers. The vertical red line indicates the moment of school dropout.

Additionally, we zoomed in on the adherence behavior of the students who dropped out of their educational trajectory, see also Figure 6. We can see two distinct patterns for the two types of dropouts: the school leavers and the switchers. School leavers show a completion percentage over time that is similar to that of the larger at-risk student group, although perhaps surprisingly, they seem to complete more than average questionnaires in the beginning of the year. It is also interesting to see that the majority of school leavers tend to keep participating in our study even after the moment of school dropout. This is different for the switchers, they participate a little less than the larger at-risk group in the beginning of the year, and their participation in our study declines sharply in the 15 weeks before the school dropout moment.

#### 5.3.2. Questionnaire Completion Times

**Figure 7:**
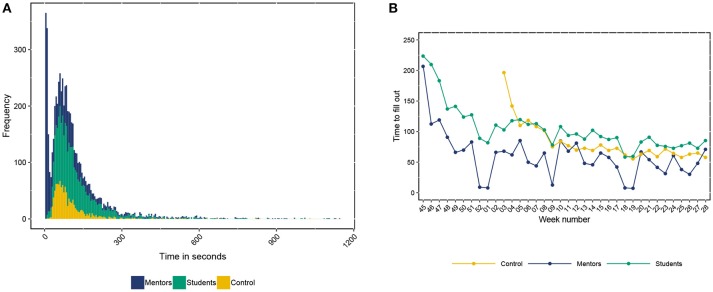
Study adherence for each subgroup. **(A)** The amount of time spent for each questionnaire in the study. **(B)** Median time spent on the questionnaires over time.

We investigated the time it takes to complete each question, and the questionnaire as a whole. The average completion time for each of the questions for both students and mentors is shown in Tables S2, S5. Figure 7A shows the distribution of completion times as measured over the whole study (i.e., the time it takes to fill out a questionnaire). Very often (in 93.65% of the cases), the questionnaire was completed within 5 min. Since there is a bimodality in the completion times, we calculated the mode for both peaks in the histogram. The first mode is 7 s, which can be explained by a mentor answering that he or she had not seen the student that week. The second mode in the data is 67 s. Mentors and at-risk students had similar completion times, while the control group generally spent less time on the questionnaire. This can be explained by the fact that the control group usually had a questionnaire with fewer questions (control = 19 vs. at-risk = 25, see also section 4.2). Figure 7B shows how the time to complete a questionnaire fluctuates over time. There is a steep decline in completion time in the first 2 to 7 weeks, perhaps indicative of a learning curve. After this the completion times become more stable, although they do mildly and gradually decline even further.

### 5.4. User Experience

As part of the post-questionnaire, we asked both of the student groups and the mentor group to evaluate the u-can-act platform. This questionnaire was completed by 59 at-risk students, 26 control students, and 12 mentors. The control group graded the platform high with an 8 (median = 8.5, range = 5–10, SD = 1.27), as did the at-risk students with a 7.84 (median = 8, range = 3–10, SD = 1.4) and the mentors with a 7.08 (median = 7, range = 5.5–9, SD = 0.93). The control group judged it to be easy to adhere to the protocol with a mean score of 79.42 (median = 83, range = 0–100, SD = 24.34), as did the at-risk students with a mean score of 73.27 (median = 81, range = 0–100, SD = 28.58) and the mentors found it more difficult than the students with a mean score of 45.67 (median = 45, range = 23–78, SD = 16.93).

## 6. Discussion

The platform that we have developed within u-can-act seems to be successful in collecting multi-informant and dynamic time-series data on within individual processes among students in vocational education—both regular students and students at risk for early school leaving—and their mentors. This is firstly evidenced by the satisfactory results of the dynamics of our EMA items: their sufficient fluctuation over time, which was on average 16.22 (in terms of RMSSD). The success of our set of innovations is furthermore evidenced by the high participant adherence among a presumably difficult target group: 68.25%. For the control group, the adherence was even higher (83%), signifying the difficulty of the at-risk subgroup (the at-risk students) we are dealing with, while adherence was lowest among the mentors (52.28%). Interestingly, among students who dropped out of their educational trajectory, the school leavers in our sample participated at the same level or even more than the at-risk group, while the switchers participated less. In addition, the questionnaire items took a relatively short time to answer, which was generally less than 5 min for the whole questionnaire. Moreover, the participants were satisfied with the user experience of the app, and indicated that it was easy to adhere to the protocol for an extended period of time, although the mentors experiences more difficulties in this. We will argue that all our (technological) innovations have contributed to these successes.

First of all, the development of the web-based platform and its innovations was essential for participation. This platform resulted in a flexible data-collection application that can be incorporated in students' daily lives by using their own smartphones. The use of a responsive web application had three major advantages: (i) the questionnaires could be filled out on any smart-phone (independent of its operating system), (ii) participants did not need download an app, and (iii) it gave mentors the option to fill out the questionnaires on a PC or tablet. Our platform was designed in such a way that it can automatically remind participants to fill in their questionnaires, to further facilitate easy participation and improve adherence. Another facilitating feature of our platform was the use of identification tokens, which meant that the participants did not need to log in (and thus did not need to remember their credentials).

We hypothesize that the high adherence is also largely influenced by our measurement protocol aimed at maximizing adherence. Because we optimized the usability of the web application by performing an elaborate quantitative and qualitative pilot study, irritations with both the technology and the formulation of the questions were discovered and solved. This led not only to high adherence, but also to a pleasant user experience which we believed helped the users of our platform to participate seriously in our study and improve the validity of their answers. We applied both internal and external motivational strategies to facilitate adherence and generate a pleasant user experience. However, we assumed that it would be unrealistic to solely rely on the intrinsic motivation for adherence of the at-risk students. We mainly dealt with adolescents at risk for early school leaving who, according to literature and our own theoretical model (see section 2) are likely to have trouble with their intrinsic motivational resources for school-related activities (Hardre and Reeve, 2003), which could affect research participation. We fostered intrinsic motivation as much as we considered possible. We used personalized messages in our invitations that were adapted to their participation behavior for example by complementing them on a long streak of filling in the app (fostering the experiences competence) and emphasized our gratefulness for their contribution to both us researchers and their mentor (fostering relatedness). We also used the name of their mentor (agency) in the application to increase the personal relevance. Apart from focusing on intrinsic motivation, we also stimulated their extrinsic motivation, by designing a monetary reward system that uses gamification and playful design elements in the form of bonus-streaks^7^. As has also been found in literature (e.g., Cerasoli et al., 2014), the combination of extrinsic motivational strategies with intrinsic motivational strategies may help foster motivation more than relying solely on intrinsic motivational strategies when it concerns simple tasks (such as filling out a questionnaire).

In the mentor study, we did not have any extrinsic motivational strategies in place, and fostered only intrinsic motivation through the same type of personalization as we did for the students. We made the assumption that their intrinsic motivation would be strong as our research would be of direct importance for the mentoring agency that they were part of, as it would provide them with important information regarding the effectiveness of the actions they take to prevent early school leaving. However, as mentor participation was quite low compared to student participation (mentors: 52.28% vs. at-risk students: 68.25% vs. control students: 83%) and the mentors indicated to experience a medium degree of difficulty in adhering to the protocol, we believe relying solely on their intrinsic motivation was insufficient. We suspect that using extrinsic motivation as a supplement (e.g., a reward system similar to that of the students) may have been helpful and consider this a promising avenue to explore in future research. Furthermore, the relatively low mentor participation may also be improved by re-evaluating the content of the mentor questionnaire. This questionnaire has a qualitative part where mentors fill in the actions that they took in guiding their students, and to next place these actions in suitable categories. This may have been a relatively hard task for some mentors and future studies may look into how this measurement can be made easier.

We believe that the mentor-student connection was essential for study adherence, and was key to get the at-risk students to participate. We approached the at-risk students through their mentor: if students participated, they did so at the behest of their mentor. Furthermore, during the study, the mentors could monitor their students' study adherence, which allowed them to targetedly motivate each student when needed to increase adherence.

### 6.1. The Innovations Have Produced an Open Source Platform That Collects Multi-Informant Time-Series Data

In order to allow other agencies and researchers to use the u-can-act platform for their own purposes, we released it as open source software. The open source philosophy has several benefits, such as the verifiability of the source code (anyone can inspect the code and verify its logical integrity) and the fact that the software is freely available. The software package includes technical instructions on the use of the software, making it re-usable for interested others. This may be interesting for other researchers or practitioners specifically interested in processes of early school leaving and its prevention. However our platform also serves a broader audience due to its generic implementation. The u-can-act platform can be used by anyone interested to gain insight into within-individual processes and the dyadic interactions or interventions that influence these processes.

Apart from the software being freely available and verifiable, its open source availability could also attract other developers to work on the platform and maintain it past the span of the u-can-act project itself. Maintenance is crucial in a software project in order for it to remain secure and to incorporate updates of external dependencies.

### 6.2. Limitations

The u-can-act project is an important step to help reduce early school leaving. However, in the present work, we do not yet propose the means to reduce early school leaving. This was not the focus of the present paper. In this paper we aimed to present the platform that we use to collect data about the mentoring process and the process of early school leaving. Our goal with this platform is to generate knowledge that will help reduce early school leaving, but the platform is not by itself meant to directly contribute to this. This may be a direction for future research however, as the current platform can be augmented with a more elaborate dashboard for mentors, on which they could follow the development of their students and adjust their intervention accordingly. The open source nature of our platform allows for such an augmentation to be developed in the future. By presenting our design, platform and initial findings, we have taken a first step in such a direction. And even if this does not happen, we believe that the data that this platform allows us to collect will foster new insights in the individual processes surrounding early school leaving and will eventually help mentors interact with their students in such a way that early school leaving is reduced.

In u-can-act, we focused on a specific subset of the Dutch educational system: vocational education. The reason for this focus is because most early school leaving takes place in this part of the educational system, and moreover, it comprises the largest number of people in the Netherlands. Because of this specific focus, data collected in this study will only be partly generalizable. On the other hand, we argue that generalizing these data might not be useful regardless of the data collected, as in this paper we strongly advocate for a more personalized approach to dealing with early school drop out.

The software is currently in a state where it requires considerable technical expertise to tailor the platform to the needs of a new research group or mentor agency. Setting up the platform requires a few technical steps, such as setting up a server and hosting a database. We have tried to make this as easy as possible with an elaborate manual^8^ that is added to the u-can-act web application (Emerencia et al., 2017). Alternatively, the technical implementation and maintenance could be done by a professional company, but then costs would be involved. Thus, even though it is open source, the current platform is like any other questionnaire platform in the sense that it needs expertise to set-up or maintain, or requires costs for external parties to do so. We will leave it up to future researchers to decide. We are currently working on an interface so that researchers can do as much as possible of the set-up themselves to help overcome this limitation.

### 6.3. Future Research

In our future work, we aim to provide a solid understanding of early school leaving and methods to prevent this. We aim to test the theoretical model that served as the foundation of the present work. Our studies will include a mapping and profiling of student processes of dropout and persistence, and mentoring processes over time. Moreover, we will investigate the micro-level dynamics within the student, and between the student and the relevant contexts, such as the mentor, school, and non-school context. Our further research will contribute to a better understanding of the process of early school leaving and the prevention of early school leaving.

## 7. Conclusion

The present work set out to describe and evaluate a novel platform, and its technological innovations, that we have developed in our project u-can-act. The platform allows researchers to investigate within-individual processes of early school leaving and interventions in this process. In fact, with some adaptation, the platform can be useful in any situation where insight is needed in within-individual processes and the way that interventions may affect such a process. The rich and unique dataset that we collected with the u-can-act platform allows us to answer many questions related to an individualized perspective on motivation and early school dropout, which were impossible to answer without these data. Moreover, the open source nature of our platform allows other interested agencies or researchers to also collect detailed multi-informant EMA data to better understand within-individual change processes and the effects of interventions.

## Ethics Statement

The u-can-act research protocol was assessed and approved by the ethical committee of the University of Groningen under code 16351-O. All participants provided their informed consent online. No explicit informed consent was collected from the parents/legal guardians of non-adult participants, as all participants were above the age of sixteen.

## Author Contributions

FB: wrote the initial draft of the manuscript, performed the analysis. MG: wrote the initial draft of the manuscript, did the literature study. NS: performed major revisions, contacted the participating agencies/students, did the literature study. AE: performed major revisions on the manuscript, performed the analysis. EK: performed major revisions on the manuscript, supervised the implementation of the study and the u-can-act platform. PJ: performed major revisions on the manuscript, supervised the implementation of the study and the u-can-act platform.

### Conflict of Interest Statement

The authors declare that the research was conducted in the absence of any commercial or financial relationships that could be construed as a potential conflict of interest.

## Footnotes

^1^Early school leaving is defined as individuals aged 18–24 who completed at most lower secondary education (International Standard Classification of Education level 2) and who are not involved in further education or training (European Commission, 2013).

^2^Website: https://rubyonrails.org

^3^Website: https://opensource.org/licenses/MIT

^4^Website: https://app.u-can-act.nl/dummy/student

^5^Website: https://app.u-can-act.nl/dummy/mentor

^6^Thus the participants that provided informed consent.

^7^We did, however, limit the use of gamification elements in order to prevent the application as being seen as “childish.”

^8^Available online from: http://u-can-act.com
