# Supplementary material for: The u-can-act Platform: A Tool to Study Intra-individual Processes of Early School Leaving and Its Prevention Using Multiple Informants
Source: Front Psychol. 2019 Sep 20;10:1808. doi: 10.3389/fpsyg.2019.01808 (PMC6764284; doi:10.3389/fpsyg.2019.01808)
Supplement: Supplementary file 1 [file Data_Sheet_1.pdf]

frontiers

Supplementary Material: the u-can-act platform: a tool to study intra-individual processes of early school leaving and its prevention using multiple informants

1 APPLICATION DESIGN EXAMPLE

An example of the playful design and gamification elements of our approach is shown in S1b and S1c.

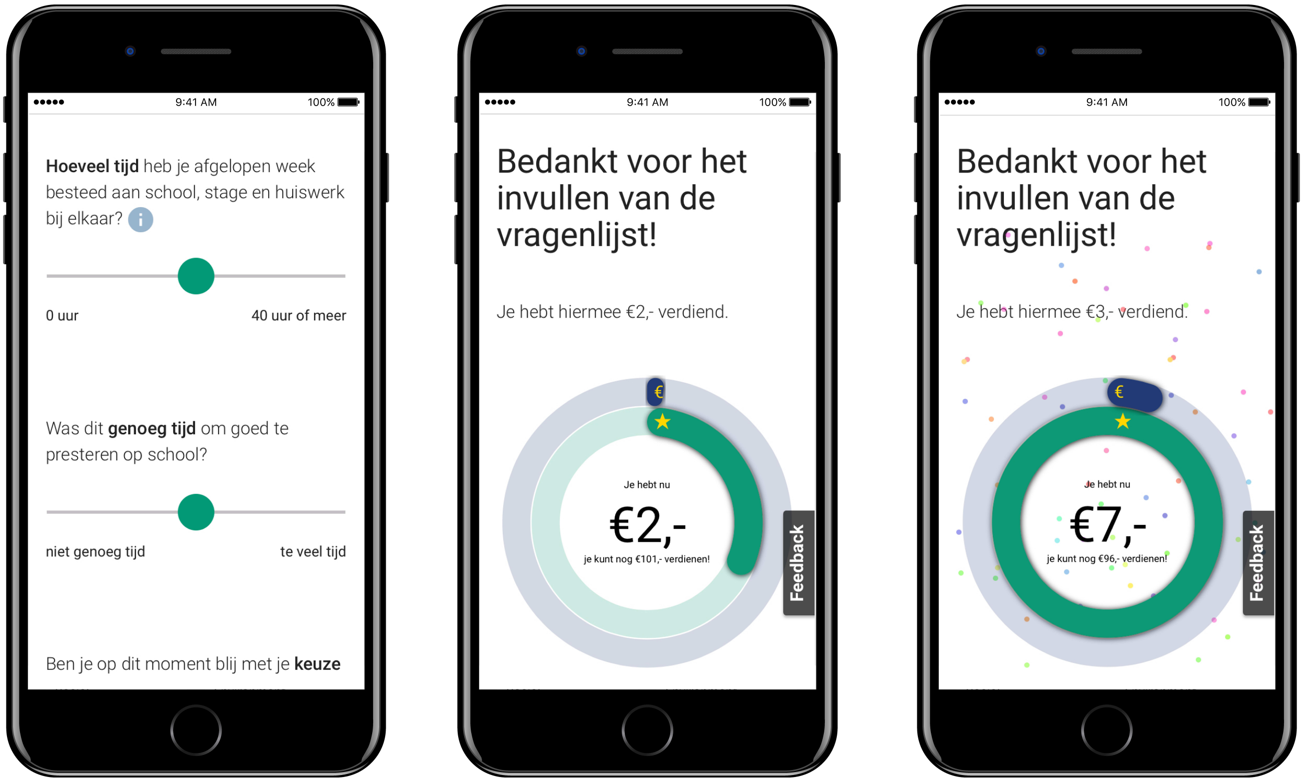

(a) Questionnaire screen. (b) Rewards screen. (c) Bonus rewards screen.

Figure S1: Screenshots of the u-can-act student web application. The (Dutch) text reads ‘Thank you for filling out the questionnaire! You earned € 2.– with this.’ The small dots on the bonus rewards screen are animated to look like fireworks. In this reward page, the students could view their progress towards the end-goal (outer ring) as well as their progress towards a ‘bonus streak’ (inner ring).

2 MENTOR DASHBOARD

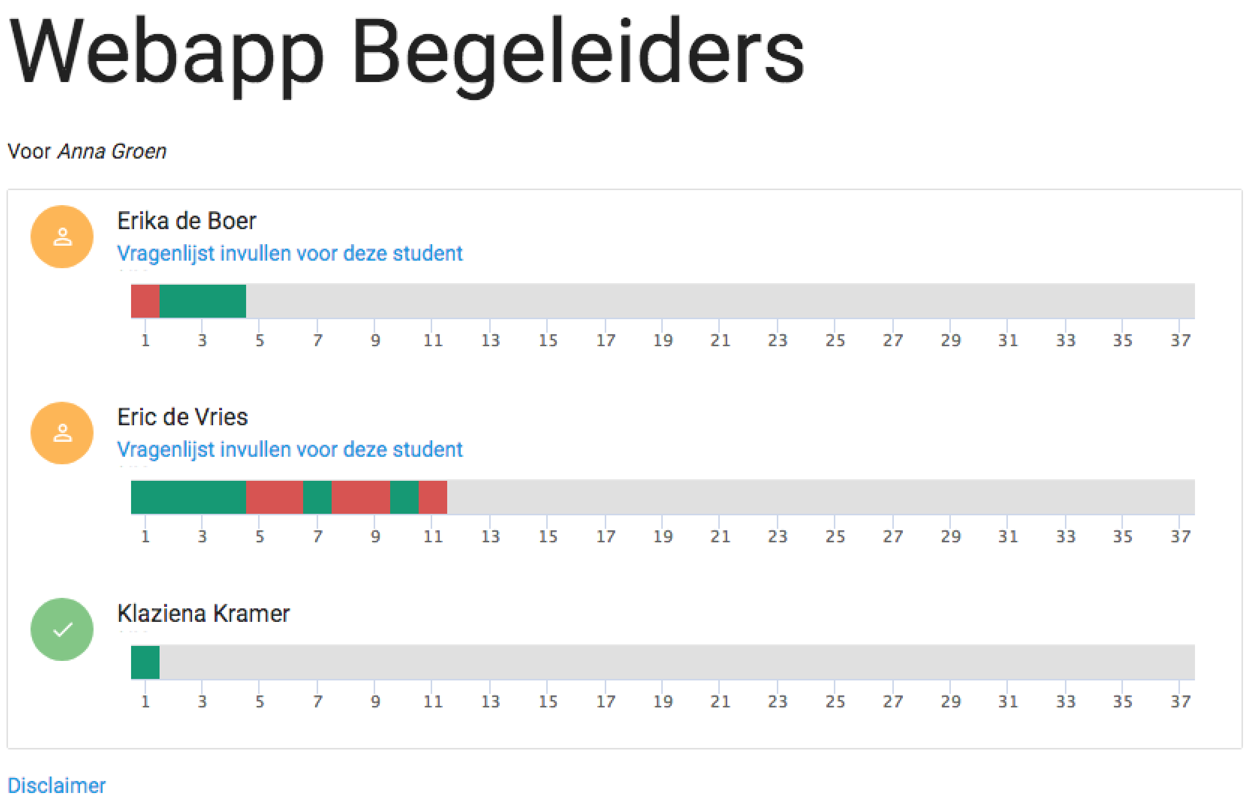

Figure S2: Illustration of the mentor dashboard. A green square represents a completed questionnaire, a red square a missed one. The blue text is a link to the questionnaire of that particular student and reads: ‘Complete the questionnaire for this student’.

3 SMS TEXT PROCEDURE

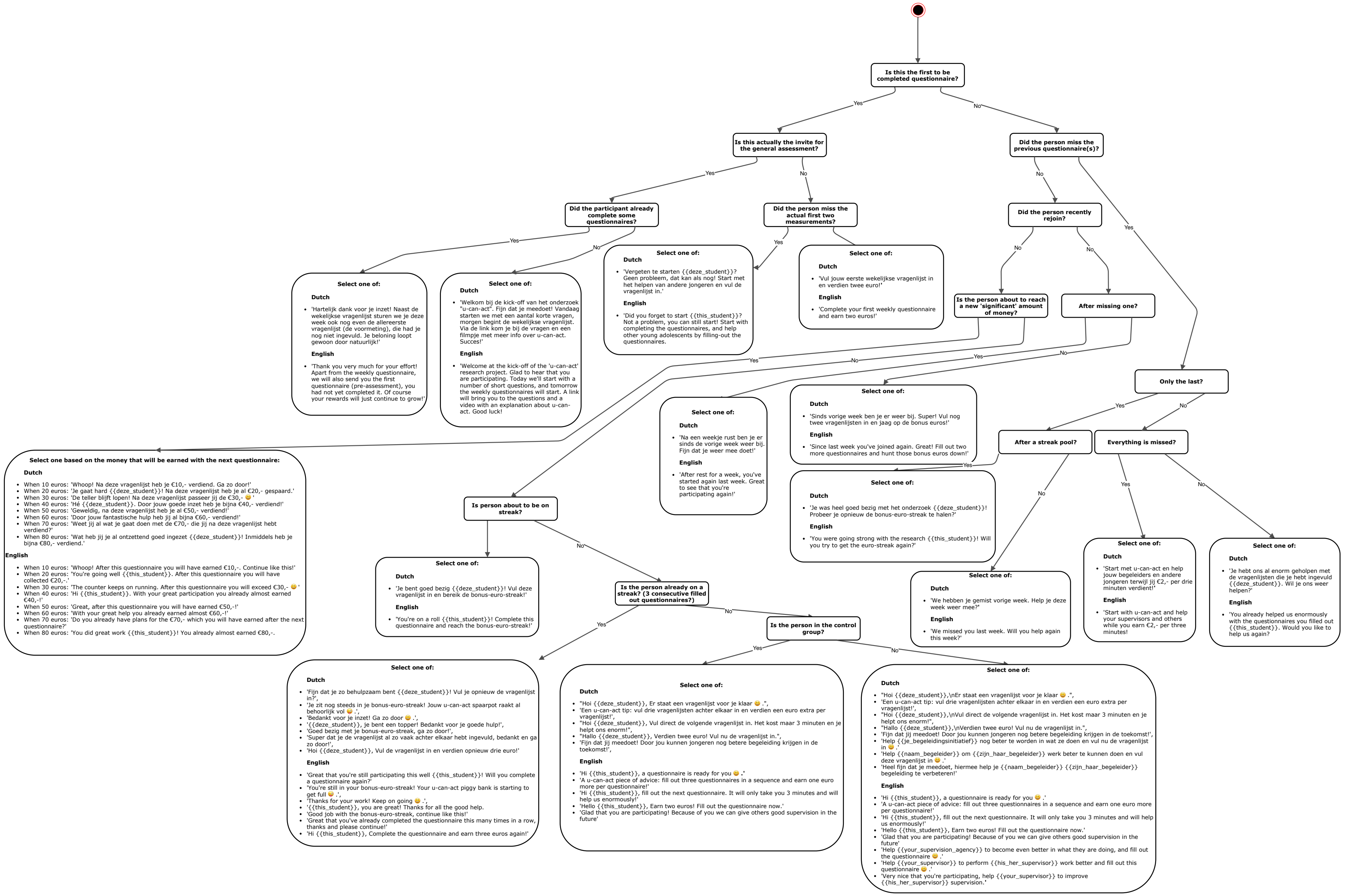

Figure S3: Determining the motivational invitation text used in SMS messages for inviting students to fill out their weekly EMA questionnaire.

4 STUDENT ASSESSMENT QUESTIONS

4.1 General assessment

Table S1: Items of the student general questionnaire.

Q	Question (Dutch)	Question (Translated)	Response range	Range	Description
1	Wat is je geboortejaar?	What is your year of birth?	Free text	Free text	Year of birth
2	Wat is je nationaliteit?	What is your nationality?	Free text	Free text	Nationality
3	Heb je op dit moment een partner?	Are you in a relationship right now?	'No, I don't have a partner', 'Yes, I have a partner', 'It is complicated', 'I'd rather not say'	Options	Relationship status
4	Heb je op dit moment kinderen?	Do you have children right now?	'No', 'Yes' (toggles optional number)	Options	Children
5	Wat is de naam van je school?	What is the name of your school?	Free text	Free text	Name of school
6	Welke opleiding doe je daar?	Which education are you enrolled in?	Free text	Free text	Name of studies
7	Op welk niveau is deze opleiding?	What is the level of this education?	'Vocational education level 1', 'Vocational education level 2', 'Vocational education level 3', 'Vocational education level 4'	Options	Level of studies
8	Hoeveel jaar ben je inmiddels bezig met deze opleiding?	How many years are you currently studying here?	Free text	Free text	Years of education at this studies
9	Wat deed je voordat je aan deze opleiding begon?	What did you do before you started with tthis education?	'Work', 'High school', 'Different vocational education'	Options	Previous occupation

4.2 Ecological momentary assessment

Table S2: Items of the student EMA study.

Q	Question (Dutch)	Question (Translated)	Response range	Range	RMSSD	Time	Description
1	Ben je de afgelopen week naar school en/of stage geweest?	Did you go to school and/or internship last week?	‘Yes’, ‘No’	Options	NA	5.99[a], 6.46[c]	School presence
2	Wat heb je de afgelopen week **meegemaakt op school en/of stage**?	What did you experience last week during school and/or internship?	‘Mostly negative things’ to ‘Mostly fun things’	0 - 100	16.4[a], 13.53[c]	5.3[a], 4.54[c]	Perception of school experiences
3	Heb je afgelopen week meestal **dingen op school en/of stage gedaan omdat** je het moest of omdat je het zelf wilde?	Did you perform the tasks at school and/or internship last week because you had to, or because you wanted to?	‘Because I had to’ to ‘Because I wanted to’	0 - 100	20.84[a], 15.71[c]	5.69[a], 5.25[c]	Autonomy vs controlled motivation
4	Hoe goed heb je dingen gedaan op school en/of stage afgelopen week?	How well did you perform your tasks at school and/or internship last week?	‘Very bad’ to ‘Very well’	0 - 100	16.02[a], 12.01[c]	5.33[a], 4.43[c]	Competence at school
5	Kon je afgelopen week goed **opschieten met vrienden op school en/of stage**?	Could you get along with your friends from your school and/or internship last week?	‘Very bad’ to ‘Very well’	0 - 100	14.04[a], 11.64[c]	4.75[a], 3.79[c]	Relatedness in relation to friends
6	Kon je afgelopen week goed **opschieten met leraren op school en/of begeleiders op stage**?	Could you get along with your teachers from your school and/or internship last week?	‘Very bad’ to ‘Very well’	0 - 100	13.72[a], 12.39[c]	4.37[a], 4.19[c]	Relateness in relation to supervisors
7	Hoeveel tijd heb je afgelopen week besteed aan school, stage en huiswerk bij elkaar?	How much time did you spend at school, internship, or on homework (summed together) last week?	‘0 hours’ to ‘40 or more hours’	0 - 100	29.51[a], 27.31[c]	5.3[a], 4.54[c]	Time spent on schoolwork
8	Was dit **genoeg tijd** om goed te presteren op school?	Was this enough time to function well?	‘Not enough time’ to ‘Too much time’	0 - 100	23.75[a], 20.21[c]	5.41[a], 4.99[c]	Time assessment

Continued on next page.

Table S2 – continued from previous page.

Q	Question (Dutch)	Question (Translated)	Response range	Range	RMSSD	Time	Description
9	Ben je op dit moment blij met je **keuze voor deze opleiding**?	Are you currently satisfied with your choice to do this study?	'Not happy with my choice' to 'Very happy with my choice'	0 - 100	13.91[a], 10.91[c]	5.21[a], 4.06[c]	Satisfaction with study choice
10	Vind je op dit moment dat je **opleiding** bij je past?	Do you think this study suits you?	'Does not fit well' to 'Fits very well	0 - 100	12.99[a], 9.24[c]	3.64[a], 3.35[c]	Subjective suitability of the study
11	Heb je er op dit moment vertrouwen in dat je dit **schooljaar gaat halen**?	Do you feel like you will pass this this year?	'No trust' to 'A lot of trust'	0 - 100	16.31[a], 11.73[c]	4.08[a], 3.85[c]	Study expectancy
12	Wat heb je de afgelopen week **meegemaakt buiten school**?	What did you experience last week outside of school and/or internship?	'Mostly negative things' to 'Mostly positive things'	0 - 100	22.56[a], 14.72[c]	5.59[a], 4.89[c]	Perception of outside of school experiences
13	Waar hadden de belangrijkste gebeurtenissen mee te maken? Je mag meerdere antwoorden geven.	What did the most important events relate to? Multiple answers are allowed	'Hobby or Sports', 'Work', 'Friendship', 'Romantic relationship', 'Home situation', 'Other:'	Options	NA	2.72[a], 2.01[c]	Important events outside of school
14	Heb je afgelopen week de meeste **dingen buiten school gedaan omdat** je het moest of omdat je het zelf wilde?	Did you perform the tasks outside of school and/or internship last week because you had to, or because you wanted to?	'Because I had to' to 'Because I wanted to'	0 - 100	20.06[a], 15.71[c]	6.32[a], 5.33[c]	Autonomous or controlled motivation
15	Hoe goed heb je dingen gedaan buiten school afgelopen week?	How well did you perform your tasks outside of school and/or internship last week?	'Very bad' to 'Very well'	0 - 100	17.78[a], 11.62[c]	4.91[a], 3.81[c]	Competence outside of school

Continued on next page.

Table S2 – continued from previous page.

Q	Question (Dutch)	Question (Translated)	Response range	Range	RMSSD	Time	Description
16	Kon je afgelopen week meestal goed **opschieten met vrienden buiten school**?	Could you get along with your friends from outside of school and/or internship last week?	'Very bad' to 'Very well'	0 - 100	15.6[a]; 10.2[c]	4.53[a]; 3.74[c]	Relatedness in relation to friends outside of school
17	Kon je afgelopen week meeestal goed **opschieten met ouders/familie buiten school**?	Could you get along with your parents / family from outside of school and/or internship last week?	'Very bad' to 'Very well'	0 - 100	16.3[a]; 11.72[c]	4.55[a]; 3.59[c]	Relatedness with respect to family
18*	Heb je de afgelopen week `naam_begeleider` gesproken?	Did you speak `mentor_name` last week?	'Yes', 'No', 'Other:'	yes / no / other	NA	4.73[a]	Mentor contact
19*	Kon je afgelopen week goed **opschieten met `naam_begeleider`**?	Could you get along with `mentor_name` last week?	'Very bad' to 'Very well'	0 - 100	7.85[a]	3.75	Relation with mentor
20*	Hoe open was je **in wat je vertelde** aan `naam_begeleider` afgelopen week?	How open were you in what you told `mentor_name` last week?	'Closed' to 'Open'	0 - 100	9[a]	3.7[a]	Openness towards mentor
21*	Heeft `naam_begeleider` je goed geholpen afgelopen week?	Did `mentor_name` help you last week?	'Did not help well' to 'Did help very well'	0 - 100	8.73[a]	3.67[a]	Effectiveness of supervision
22*	In hoeverre voelde jij je afgelopen week gesteund door `naam_begeleider` in het maken van je eigen beslissingen?	To what extent did you feel supported by `mentor_name` in making your own decisions last week?	'Not at all' to 'Very strongly'	0 - 100	9.41[a]	4.36[a]	Experience of autonomy support
23*	In hoeverre had jij het gevoel dat `naam_begeleider` er voor je was deze week?	To what extent did you have the feeling `mentor_name` was there for you?	'Not at all' to 'Very strongly'	0 - 100	8.72[a]	4.38[a]	Experience of relatedness support

Continued on next page.

Table S2 – continued from previous page.

Q	Question (Dutch)	Question (Translated)	Response range	Range	RMSSD	Time	Description
24[*]	In hoeverre gaf `naam_begeleider` je afgelopen week het gevoel dat je dingen goed kan?	To what extent dit `mentor_name` give you the feeling you can perform your tasks well?	‘Not at all’ to ‘Very strongly’	0 - 100	8.17[a]	4.29[a]	Experience of competence support
25	Hoe voelde jij je deze week?	How did you feel this week?	‘Very bad’ to ‘Very well’	0 - 100	21.73[a], 15.65[b]	5.26[a], 4.47[c]	Moment-to-moment quality of life

Notes: The text written in the fixed width font are replaced with their actual contents.

[a] At-risk group.
[c] Control group.
[*] Only available in the at-risk students’ questionnaire.

4.3 Post-assessment

Table S3: Items of the student post questionnaire.

Q	Question (Dutch)	Question (Translated)	Response range	Range	Description
1	Ben je dit schooljaar definitief gestopt met je opleiding?	Did you permanently stop your education this schoolyear?	'Yes, I stopped without graduating' (shows questions 2, 3 and 4), 'Yes, I graduated' (shows questions 2 and 4), 'No I did not stop' (shows question 5)	Radio	Indication of stopping education
2[a]	Wanneer ben je ongeveer gestopt? Als je het niet precies meer weet, vul dan iets in dat zo goed mogelijk in de buurt komt.	When did you approximately stop? If you do not know exactly, please provide the date that approximates the actual date the best.	Date	Date	Determine when stopped
3[a]	Hoeveel jaar moest je nog ongeveer tot je diploma?	How many years of education did you have left before the expected graduation year?	'0 to 1 year', '1 to 2 years', '2 to 3 years', '3 years or more'	Options	Length of education remaining.
4[a]	Ben je van plan om met een nieuwe opleiding te starten?	Do you plan to start with a new studies?	'Yes', 'No', 'I'm not sure'	Options	Future study plans
5[a]	Hoe zeker ben je ervan dat je jouw opleiding gaat afmaken?	How certain are you that you will finish your studies?	'Not sure at all' to 'Totally sure'	0-100	Certainty of finishing studies
6	Wat voor cijfer sta/stond je gemiddeld voor al je vakken samen?	What was your average grade for all courses?	'1' to '10', steps of .5. When unable to answer this question, question 6.1 was shown	1-10	Average performance on all subjects
6.1[a]	Kun je iets zeggen over hoe je ervoor staat/stond qua cijfers?	Can you give a general impression on how you are currently doing with respect to your grades?	'Very bad' to 'Very good'	0-100	General impression of study performance

Continued on next page.

Table S3 – continued from previous page.

Q	Question (Dutch)	Question (Translated)	Response range	Range	Description
7*	Vind je dat de begeleiding die jij krijgt van `je_begeleidings-initiatief` bij de school hoort, of juist los staat van de school?	Do you have the feeling that the supervision you get from `your_supervision-agency` is part of school, or is actually a separate entity?	'Totally a part of school' to 'Completely separate from school'	0-100.	Judgment of the supervision context
8*	Hoe zou je de begeleiding die je van je_begeleidingsinitiatief hebt gekregen in één woord beschrijven?	How would you describe `your_supervision-agency` in one word?	Free text	Free text	General description of supervision agency
9*	Hoe nuttig vond jij de begeleiding die je hebt gekregen van `je_begeleidings-initiatief`?	How useful did you find the supervision `your_supervision-agency` provided?	'Not useful at all' to 'Very useful'	0-100	Judgment of usefulness of supervision
10*	Wil je je ingevulde vragenlijsten delen met `naam_begeleider`, zodat hij/zij ervan kan leren? Als je nee aanvinkt krijgt `naam_begeleider` jouw ingevulde vragenlijsten niet te zien.	Would you like to share your questionnaires with `your_supervisor` for him or her to learn from it? If you select no, `your_supervisor` won't be able to see your questionnaires.	'Yes', 'No'	Options	Willingness to share answers.
11	Hoe vond jij het om ongeveer een half jaar lang de webapp wekelijks in te vullen?	How much did you like filling out the web application for half a year?	'Very difficult to maintain' to 'Very easy to maintain'	0-100	Experienced difficulty of participation.
12	Wat voor cijfer zou je de webapp geven?	Which grade would you give the web application?	'1' to '10', steps of .5	1-10	Appreciation of the web application
13	Zou je jouw vrienden aanraden om ook mee te doen aan het u-can-act onderzoek?	Would you recommend your friends to partake in the u-can-act research project?	'No not at all' 'Yes definitely'	0-100	Appreciation of web application with respect to friends

Continued on next page.

Table S3 – continued from previous page.

Q	Question (Dutch)	Question (Translated)	Response range	Range	Description
14	Heb je nog tips voor ons om het onderzoek of de webapp beter te maken in de toekomst?	Do you have any advice for us to improve the web application for future projects?	Free text	Free text	Room for improvement

Notes: The text written in the fixed width font are replaced with their actual contents.

[a] Hidden by default.

[*] Only available in the at-risk students' questionnaire.

5 MENTOR ASSESSMENT QUESTIONS

5.1 General assessment

Table S4: Items of the mentor general questionnaire.

Q	Question (Dutch)	Question (Translated)	Response range	Range	Description
1	Wat is jouw hoogst genoten opleiding?	What is your highest level of education?	‘No education’, ‘Primary school’, ‘Primary or ‘preparational vocational education’, ‘Secondary education’, ‘Middle level applied training’, ‘Higher secondary education’, ‘Higher applied education’, ‘Scientific education / University’	Options	Education level
2	Wat is je geboortejaar?	What is your year of birth?	Free text	Free text	description
3	Hoeveel jaar heb jij tot nu toe gewerkt in de jongeren-begeleiding?	How many years have you been working in supervising adolescents?	Free text	Free text	description
4	Wat is je nationaliteit?	What is your nationality?	Free text	Free text	description

5.2 Ecological momentary assessment

Table S5: Items of the mentor EMA study.

Q	Question (Dutch)	Question (Translated)	Response range	Range	RMSSD	Time	Description
1	Heb je deze week acties ondernomen in de begeleiding van deze_student?	Did you perform any actions in the supervision of this_student?	'Yes' (shows questions 3, 4, 5, 6, 7, 8, and 9), 'No' (shows question 2)	Options	NA	3.64	Performed actions
2	Waarom heb je deze week geen acties ondernomen in de begeleiding van deze_student?	Why did you not perform any actions regarding the supervision of this_student?	'I did not have contact with this_student', 'I stopped supervising this_student' (shows questions 14 and 15), 'this_student dropped out' (Shows questions 16, 17, 18), 'I transferred the supervision of this_student to someone else' (shows questions 10, 11, 12, 13)	Options	NA	5.35	Reason for not performing actions
3	Nog een actie(reeks) toevoegen / Verwijder actie(reeks)	Add another set of actions / remove actions	See Table S6	NA	NA	NA	Actual performed actions
4	Hoeveel tijd heb je deze week besteed aan de begeleiding van deze_student?	How much time did you spend supervising this_student this week?	Hours and minutes	0-11 (hours), 0 – 60 (minutes, steps of 15 minutes)	68.47	NA	Time spent supervising

Continued on next page.

Table S5 – continued from previous page.

Q	Question (Dutch)	Question (Translated)	Response range	Range	RMSSD	Time	Description
5	Waren jouw acties in de begeleiding van deze_student deze week vooral gepland of vooral intuïtief?	Were your actions regarding the supervision of this_student mainly intuitive or planned?	'Totally intuitive' to 'Planned in advance'	0-100	23.82	13.76	Planned or intuitive actions
6	In hoeverre heb jij deze week geprobeerd deze_student te ondersteunen in het maken van zijn_haar_student eigen beslissingen?	To what extent did you try to support this_student in his_or_her own decisions?	'Not' to 'Very strongly'	0-100	22.17	7.56	Support autonomy
7	In hoeverre heb jij deze week geprobeerd deze_student het gevoel te geven dat hij_zij_student dingen goed kan?	To what extent did you try to give this_student the feeling that he_or_she is competent?	'Not' to 'very strongly'	0-100	19.97	6.01	Support competence
8	In hoeverre heb jij deze week geprobeerd deze_student het gevoel te geven dat je er voor hem_haar_student bent?	To what extent did you try to give this_student the feeling that you are there for him_or_her ?	'Not' to 'very strongly'	0-100	14.01	5.53	Support relatedness

Continued on next page.

Table S5 – continued from previous page.

Q	Question (Dutch)	Question (Translated)	Response range	Range	RMSSD	Time	Description
9	Heb je de begeleiding van `deze_student` deze week grotendeels overgedragen aan een andere persoon?	Did you transfer the main supervision of `this_student` to a different person?	‘Yes’ (shows questions 10, 11, 12), ‘No’	Options	NA	4.36	Transfer supervision to other mentor
10	Waarom heb jij de begeleiding (grotendeels) overgedragen?	Why did you transfer the (main) supervision?	Free text	Free text	NA	17.54	Transfer supervision to other mentor
11	Aan wie heb jij de begeleiding (grotendeels) overgedragen?	To whom did you transfer the (main) supervision?	Free text	Free text	NA	9.73	Transfer supervision to other mentor
12	Wat denk jij dat diegene deze week heeft gedaan in de begeleiding van `deze_student`?	What do you think that person did in the supervision of `this_student` this week?	Free text	Free text	NA	15.94	Transfer supervision to other mentor
13	Is de overdracht van begeleiding van permanente aard? Mail dan de telefoonnummers van jou, `deze_student` en de nieuwe begeleider.’	Is this supervision transfer permanent? (If so, send an email with the details about the new mentor)	NA (Notice)	NA	NA	NA	Transfer supervision to other mentor

Continued on next page.

Table S5 – continued from previous page.

Q	Question (Dutch)	Question (Translated)	Response range	Range	RMSSD	Time	Description
14	Waarom ben je gestopt met de begeleiding van deze_student?,	Why did you stop supervising this_student?	Free text	Free text	NA	22.64	Why stopped supervision
15	Denk jij dat deze_student nog steeds risico loopt om voortijdig te stoppen met zijn_haar_student opleiding?,	Do you think this_student is still at risk of early dropping out of school?	‘Yes’, ‘No’	Options	NA	6.4	Dropout risk assessment
16	Waarom is deze_student gestopt met zijn_haar_student opleiding?,	Why did this_student stop with his_or_her studies?	Free text	Free text	NA	15.17	Why student stopped
17	Wat gaat deze_student doen nu hij_zij_student gestopt is met zijn_haar_student opleiding?,	What will this_student do now after he_or_she has stopped with his_or_her studies?	‘Work’, ‘Do a different studies’, ‘I do not know’	Options	NA	5.08	Student future
18	Stopt jouw begeleiding van deze_student nu hij_zij_student met de opleiding is gestopt?	Will your supervision stop now that this_student has stopped his_or_her studies?	‘Yes’, ‘No’	Options	NA	5.71	Student stopped, supervision status

Table S6: Items of the mentor action clusters. The questions appeared after adding a new action cluster using question three of the mentor questionnaire.

Q	Question (Dutch)	Question (Translated)	Response range	Range	Description
3.1	Welke belangrijke actie, of reeks aan acties die volgens jou bij elkaar horen (bijv. omdat ze hetzelfde doel dienen of kort achter elkaar zijn uitgevoerd), heb jij uitgevoerd in de begeleiding van deze_student?	Which important action, or number of grouped actions (e.g., because they serve the same purpose or were executed with little delay) did you perform in the supervision of this_student	Free text	Free text	Goal description
3.2	In welke categorie(ën) past de zojuist beschreven actie(reeks) volgens jou het beste?	Which category (or categories) best fit this action or these actions?	'High level contact', 'Visual exercises', 'Verbal exercises', 'Motivation', 'Confronting', 'Explain', 'Support for school work', 'Provide emotional support', 'Involve this_student's social environment', 'Ask for help from colleagues or other professionals', 'Perform observations'	Options	Action categorization

Continued on next page.

Table S6 – continued from previous page.

Q	Question (Dutch)	Question (Translated)	Response range	Range	Description
3.3	Aan welke doelen heb jij gewerkt door deze actie(s) uit te voeren?	Which goals did you work on with this action?	‘Improve the relationship with this_student’, ‘Develop emotional wellbeing of this_student,’ ‘Develop skills of this_student’, ‘Enable this_student to self-reflect’, ‘Get an insight into the experiences of this_student’, ‘Get an insight into the social environment of this_student’, ‘Change the social environment of this_student’	Options	Goal categorization
3.4	Hoe belangrijk denk jij dat deze actie(reeks) was voor de voortgang van deze_student in zijn_haar_student begeleidingstraject?	How important do you think this action / these actions were this_student his_her_student progress?	‘Not important’ to ‘Very important’	0-100	Importance of action
3.5	Hoe tevreden ben je met de interactie tussen jou en deze_student?	How satisfied are you with the interaction between you and this_student?	‘Dissatisfied’ to ‘Very satisfied’	0-100	Satisfaction with interaction

Notes: The text written in the fixed width font are replaced with their actual contents.

5.3 Post-assessment

Table S7: Items of the mentor post questionnaire.

Q	Question (Dutch)	Question (Translated)	Response range	Range	Description
1	Kan je in één woord beschrijven hoe jij het ervaart om jongeren te begeleiden binnen `je_begeleidingd-initiatief` ?	Can you describe your experience about how you experience supervising students within `your_supervision-agency`?	Free text	Free text	Supervision experience
2	In hoeverre vind jij dat `deze_student` vooruitgang heeft geboekt in `zijn_haar_student` begeleidingstraject?	How much progress do you think `this_student` made during `his_or_her` supervision?	'Not at all' - 'A lot'	0-100	Question was dynamically created for each student.
3	Hielp het invullen van de webapp je om jouw jongeren beter te begeleiden?	Did the application help you for supervising your students?	'Not at all' to 'A lot'	0-100	Experience of support provided by the application
4	Hoe vond jij het om de webapp ongeveer een half jaar lang wekelijks in te vullen?	How hard did you find it to fill out the application every week for a period of half a year?	'Very hard' to 'Very easy'	0-100	Difficulty of filling out the application
5	Wat voor cijfer zou je de webapp geven?	Which grade would you give the Web application?	'1' to '10'	1-10 (steps of 0.5)	Appreciation of the application
6	Zou je jouw collega's aanraden om ook mee te doen aan het u-can-act onderzoek?	Would you advise your colleagues to participate in u-can-act?	'Not at all' to 'Definitely'	0-100	Advising other colleagues to participate
7	Heb je nog tips hoe wij het onderzoek of de webapp beter kunnen maken in de toekomst?	Do you have comments or tips for improvement?	Free text	Free text	Improvements to the application

Notes: The text written in the fixed width font are replaced with their actual contents.
